# Patient-reported impacts of a conservative management programme for the clinically inapparent adrenal mass

**DOI:** 10.1007/s12020-012-9856-z

**Published:** 2012-12-20

**Authors:** Andreas Muth, Charles Taft, Lilian Hammarstedt, Lena Björneld, Mikael Hellström, Bo Wängberg

**Affiliations:** 1 Department of Surgery, Sahlgrenska University Hospital, Sahlgrenska Academy, University of Gothenburg, SE-413 45 Gothenburg, Sweden; 2 Institute of Health and Care Sciences, University of Gothenburg Centre for Person-Centred Care, Sahlgrenska Academy, University of Gothenburg, Gothenburg, Sweden; 3 Department of Radiology, Sahlgrenska Academy, University of Gothenburg, Gothenburg, Sweden

**Keywords:** Adrenal gland neoplasm, Incidental findings, Computed tomography, Quality of life, Follow-up, Adult

## Abstract

The aim of this study was to assess patient-reported impacts and health-related quality of life (HRQL) of a 2-year follow-up programme in a large cohort of patients with stationary, non-functioning, adrenal incidentalomas (AIs) in western Sweden. 145 patients (mean age 68 years, 62 % females) with AI from a prospective study in western Sweden were studied. All had completed a 2-year follow-up programme by November 2007, without evidence of adrenal malignancy or hormone over-production. To evaluate patient-reported impacts and HRQL, an eight-item adrenal incidentaloma impact questionnaire was used retrospectively, together with the hospital anxiety and depression scale, and the short form-36. There were 111 patients (mean age 67 years, 63 % females) who responded to the questionnaire (response rate 77 %). 77 % reported that the AI diagnosis had caused them to be worried; however, fewer than 20 % had thought about the lesion often during the follow-up programme, and only 3 % had felt that it had a large impact on their current daily life. Only 4 % stated that the follow-up programme had been a negative experience, nevertheless 10 % reported a negative impact on their HRQL during the follow-up programme. Only 2 % stated that release from follow-up caused worry to any degree. In total, 29 % had possible anxiety, and 30 % had possible depression, probably reflecting significant co-morbidity. Possible anxiety correlated with a more negative experience of the follow-up programme. In conclusion, the 2-year follow-up programme for patients with AI was well tolerated. Nonetheless, a small number remained worried throughout follow-up, suggesting the need for tailored counselling in individual patients to ameliorate negative impacts of follow-up.

## Introduction

The purpose of follow-up for patients with incidentally discovered adrenal lesions (also known as adrenal incidentalomas (AIs)) is to identify individuals with malignant or hormone-producing tumours. However, the majority of patients with AIs have benign, non-functioning adenomas that can be managed conservatively [1–6]. Management of patients with AI imposes a significant burden on health-care providers, as adrenal lesions are frequent findings on abdominal computed tomography (CT) and magnetic resonance imaging performed for unrelated reasons. In a survey in western Sweden, AIs were seen in 4.5 % (range 1.8–7.1 % between hospitals) of abdominal CT scans [7]. In autopsy studies, the prevalence of adrenal tumours approaches 7 % in patients over 70 years of age [8]; hence, as advanced cross-sectional imaging is increasingly performed in the elderly population the detection of AIs can be expected to rise.

Concerns about hormone-production or malignancy developing in the AI over time provide the rationale for follow-up programmes, but the extent of follow-up is debated [9, 10]. In 2002, the U. S. National Institute of Health published a state-of-the science report on the incidentally discovered adrenal mass, identifying areas of interest for future studies [11]. The impact of being diagnosed with an AI and subsequent follow-up on health-related quality of life (HRQL) was highlighted as a priority area. To date, little research has addressed this issue.

We assessed patient-reported impact of a 2-year follow-up programme and HRQL in a large cohort of patients with stationary, non-functioning AIs in western Sweden.

## Patients and methods

### The prospective cohort study of AI in western Sweden

During an 18-month period, all patients with AIs identified at all radiology departments in western Sweden (serving in- and out-patient care for 1.7 million inhabitants,) were prospectively reported and enrolled in a 2-year follow-up programme. The programme has previously been described in detail [6, 7]. At detection, the patients were informed about the study by their attending physician, and referred for evaluation to one of the local study coordinators (consultants in internal medicine, endocrinology or endocrine surgery) at the Sahlgrenska University Hospital, or one of the six county hospitals in the region. All patients received oral and written information about the study and gave written consent. key points in the written information were that: a lesion in the adrenal had been incidentally found; adrenal lesions in most cases are benign and require no specific treatment, although some represent ‘tumours’ that may require surgery or special medication; the purpose of follow-up was to detect hormone-producing lesions or lesions suspicious for malignancy; the follow-up programme in the study was compliant with national guidelines; and if no signs of hormone production or malignancy were seen after 2 years, then no further follow-up was necessary. Additional individualized information was provided as found necessary.

### The follow-up programme

Dedicated adrenal CT was scheduled at 4, 12 and 24 months as previously described [6]. Clinical and biochemical evaluation was scheduled at baseline and after 24 months. Adrenomedullary function was assessed with 24 h urinary catecholamines and/or metanephrines. Adrenocortical function was assessed using 24-h urinary-free cortisol (UFC) and aldosterone (in hypertensive patients, measurements were taken once again); plasma aldosterone and upright renin were assessed; and a 1-mg dexamethasone suppression test (1 mg-DST) was performed. After 24 months, baseline biochemical work-up was repeated, and all patients underwent 1 mg-DST. Suppression <60 nmol/L [12] was regarded as normal, while values >138 nmol/L [13] were regarded as insufficient. In patients with 1 mg-DST cortisol levels in the range of 60–138 nmol/L, other factors such as age and co-morbidities were taken into account. Patients with insufficient suppression at 1 mg-DST were scheduled for further examinations, including measurement of ACTH, long-term suppression tests and repeat UFC.

### Criteria for conservative management

Criteria for conservative management were radiologically stationary lesions with benign features [14], and no evidence of hormone over-production (including subclinical hypercortisolism). Follow-up was terminated after 24 months in patients who fulfilled these criteria, and these patients were eligible for inclusion in the present study. Adrenalectomy was considered for patients with unilateral tumours >3 cm in transaxial diameter, tumours with interval growth, or tumours with other features suspicious for malignancy [14] and/or hormone over-production. Some patients with tumours >3 cm were managed conservatively because of benign radiological characteristics, severe comorbidity, or patient preferences.

### Study population

One hundred and forty-five patients (mean age 68 years, 62 % females), who fulfilled the criteria for conservative management and had completed the 2-year follow-up programme by November 2007, constituted the study population. Information on results of biochemical evaluations (UFC and 1 mg-DST cortisol levels), patient’s height and weight, the presence of diabetes, hypertension, other cardiovascular disease, and previous history of malignancy was retrieved from the main study database. Diagnoses of malignancy were crosschecked against the Swedish National Cancer Register (http://www.socialstyrelsen.se/register/halsodataregister/cancerregistret/inenglish).

### Development of an adrenal incidentaloma impact questionnaire (AIIQ)

In order to gain insight into the patients’ experiences of the follow-up programme, interviews were conducted by one of the authors (BW) with two randomly selected patients who had been managed conservatively for AI. Analysis of the interview material yielded a dominating theme of worry–relief. On the basis of these interviews, an 8-item questionnaire was developed with questions regarding specific points of interest during the follow-up programme. Items were constructed to assess: (A) worry, preoccupation and psychological impact of the diagnosis and follow-up of the AI, and (B) appraisals of the follow-up programme as such. Items were framed to cover: (1) the time of diagnosis, (2) the follow-up period, (3) the time at completion of the programme, and (4) the time after finishing the programme. A panel of physicians with experience in treating AI patients reviewed the items, and the questionnaire was assessed for face validity (comprehensibility, relevance and comprehensiveness) by senior nurses at the endocrine unit.

### Rationale for using additional instruments

We assumed that the follow-up programme for AI would affect the patients’ HRQL principally in the area of psychological well-being and mental health, rather than physical functioning. The Swedish version of the generic short form-36 (SF-36) [15] was used to assess HRQL. The SF-36 is a 36-item survey that measures eight domains of health: physical functioning, role limitations due to physical health, bodily pain, general health perceptions, vitality, social functioning, role limitations due to emotional problems, and mental health. An age- and sex-matched reference group (*n* = 145) was randomly drawn from the Swedish normative database [15]. To evaluate anxiety and depression, we used the hospital anxiety and depression scale (HADS) [16]. The HADS consists of two subscales evaluating anxiety (HADS-A, 7 items) and depression (HADS-D, 7 items). We used established and validated cut-off values (<8 non-cases, 8–10 possible cases, and >10 probable cases) for each scale that were proposed by the developers. Using the cut-off ≥8, the sensitivity and specificity for detecting anxiety or depression are both approximately 0.8 [17].

### Administration of questionnaires

The three questionnaires, accompanied by a letter signed by the two principal investigators of the clinical follow-up study and a stamped return envelope addressed to an independent institute, were sent by mail on the 15th of November 2007. Two reminders were used. The second reminder included a new copy of the questionnaire and patients who declined to participate were asked to endorse a reason for not responding. Response alternatives were: To my knowledge I have not participated in such a programme; I think the questionnaire is too long; I haven’t time; I don’t understand the questions; I don’t see the purpose of the questionnaire; I see no point in answering the questionnaire; I don’t think the questions are about me; I think the questions are too intimate; I don’t feel strong enough to answer the questions; Other reason, please state:….

### Statistical analysis

Frequencies were computed for each of the AIIQ items. Response alternatives were coded from 1 to 5, where higher values indicate more positive responses. Inter-item correlations were calculated using Spearman’s rho (*r*_s_). An exploratory factor analysis using Varimax rotation was performed. The Kaiser rule for factor extraction was applied. Summated scores of the items comprising the factors were correlated (*r*_s_) with the scores on the HADS and the SF-36.

Correlations between patient characteristics (age, sex and co-morbidities), tumour characteristics (tumour size and location (uni- or bilateral), UFC and 1 mg-DST cortisol levels), and HRQL were performed using Spearman’s rho. To assess the impact of co-morbidities, a crude co-morbidity index was used awarding one point each for hypertension, other cardiovascular disease (e.g. congestive heart failure and paroxysmal atrial fibrillation), diabetes and previous or concurrent of extra-adrenal malignancy, yielding a score from 0 to 4.

Analysis of AIIQ items across different participating clinical units, and comparisons between AIIQ items and HADS (non-cases, possible cases and probable cases) were performed using the Kruskal–Wallis test followed by the Mann–Whitney *U* test.

Further comparisons between HADS non-cases, possible and probable cases, and UFC- and 1 mg-DST cortisol levels were performed using one-way analysis of variance (ANOVA), and, for 1 mg-DST, the Chi-square test using serum cortisol cut-off levels of 50 [18], 60 [12], 83 [19] and 138 [13] nmol/L. SF-36 data from respondents, and matched controls was analysed using the Mann–Whitney *U* test. Calculations were performed using IBM SPSS Statistics version 19 (IBM Corporation, Somers, NY).

## Results

### Patient characteristics and response rate

In total, 111 patients (mean age 67 years; 63 % females) responded (response rate 77 %). Data were complete for the AIIQ in 110 cases (109 in items 3 and 8), HADS-anxiety (HADS-A) in 108 cases, HADS-D in 109 cases and SF-36 in 108 cases. For characteristics of responders and non-responders, see Table 1. In 15 of 34 cases, a reason for not responding was stated: Old age/Questionnaire too demanding *n* = 6; Questionnaire not relevant *n* = 3; Dementia *n* = 2; No recollection of having participated in a follow-up programme *n* = 2; Language problems *n* = 1; Matter of principle *n* = 1. In 19 cases, no explanation was offered.

**Table 1:** Characteristics of patients responding and not responding to the questionnaires

	Responders	Non-responders
Subjects, *n* (%)	111 (100)	34 (100)
Age in years, mean (median, range)	67 (67, 30–90)	70 (74, 27–89)
Females, *n* (%)	70 (63)	20 (59)
Bilateral lesions, *n* (%)	23 (21)	9 (26)
Size in mm, mean (median, range)	24 (23, 12–91)	26 (22, 15–54)
UFC at completion, mean ± 2SD (ref. 75–350 nmol/24 h)	168.8 ± 201.3	180.7 ± 143.1
1 mg-DST cortisol levels at completion, mean ± 2SD (nmol/L)	54.7 ± 63.8	49.6 ± 43.4
Diagnosis at detection*, *n* (%)		
B Infection	1 (1)	0 (0)
C Malignant neoplasm	20 (18)	2 (6)
D 00–48 Benign neoplasm	3 (3)	1 (3)
D 50–89 Blood	1 (1)	0 (0)
E Endocrine	1 (1)	0 (0)
F Mental/behavioural	0 (0)	1 (3)
I Circulatory	11 (10)	1 (3)
J Respiratory	4 (4)	2 (6)
K Digestive	36 (32)	11 (32)
M Musculoskeletal	8 (7)	0 (0)
N Genitourinary	2 (2)	2 (6)
R Symptoms	16 (14)	5 (15)
S Trauma	2 (2)	4 (12)
Y External causes	2 (2)	0 (0)
Z Control after therapy	4 (4)	5 (15)
Co-existing conditions, *n* (%)		
Hypertension	53 (48)	13 (38)
Cardiovascular disease^†^	25 (23)	6 (18)
Diabetes mellitus	10 (9)	10 (29)
Hyperlipidemia	13 (12)	1 (3)
Osteoporosis	5 (5)	3 (9)
BMI (kg/m^2^) > 25^‡^	45 (41)	7 (21)
History of malignancy	18 (16)	6 (18)

*UFC* urinary free cortisol, 1 mg*-DST* 1 mg overnight dexametasone suppression test

* Results of the work-up for the complaints that led to the incidental discovery of adrenal lesions, grouped according to the International Classification of Diseases, version 10 (ICD-10), ^†^ Other than hypertension (ICD-10 code I 10.9), ^‡^ BMI data missing on 25 responders and 15 non-responders

### Responses to the AIIQ and factor analysis

**Fig. 1:**
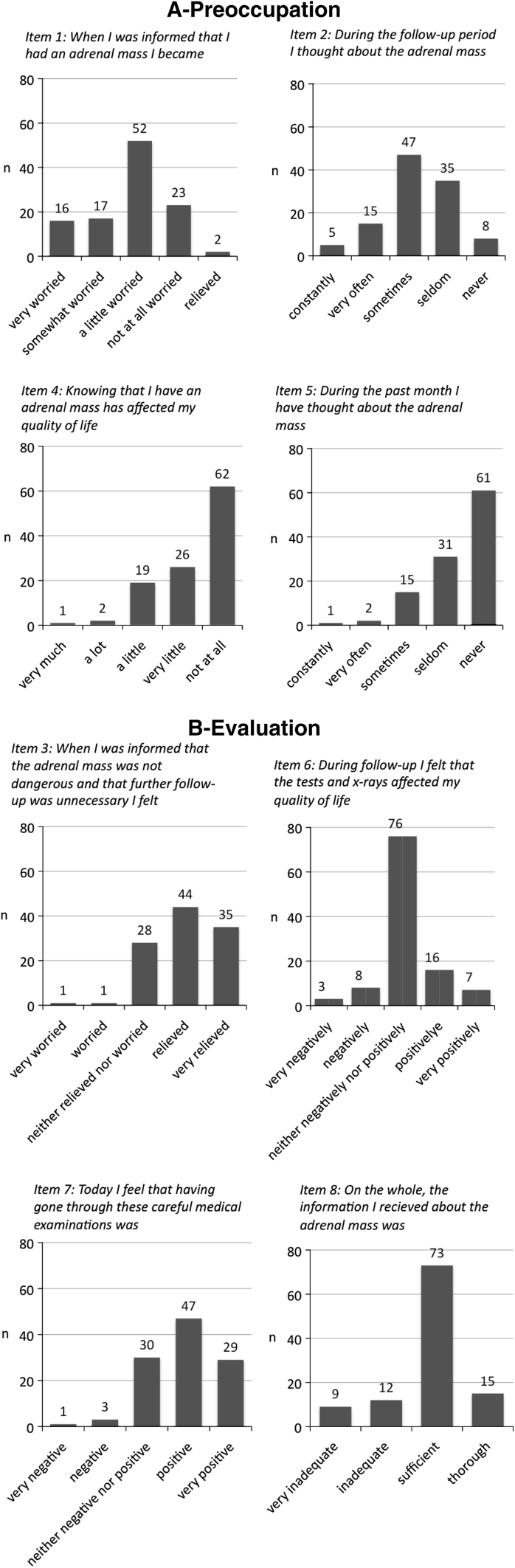
The adrenal incidentaloma impact questionnaire, distribution of responses for each item. Items grouped according to the factor analysis. **a** Preoccupation with the adrenal incidentaloma. **b** Evaluation of the follow-up programme

Responses to the AIIQ are summarized in Fig. 1a, b. Exploratory factor analysis supported a two-factor model (eigenvalue >1) explaining 61 % of the variance (Table 2). AIIQ-items 1, 2, 4 and 5 formed the first factor, labelled *preoccupation* with the AI, while the second factor comprised items 3 and 6–8, and was labelled *evaluation* of the programme as such.

**Table 2:** The adrenal incidentaloma impact questionnaire–exploratory factor analysis

	Factor	
AIIQ	Preoccupation	Evaluation
Item 1	0.843	−0.049
Item 2	0.863	−0.092
Item 3	−0.268	0.755
Item 4	0.683	0.375
Item 5	0.761	0.322
Item 6	0.267	0.577
Item 7	0.107	0.762
Item 8	0.152	0.679

Factor loadings in the final model

#### Preoccupation with the AI

The AI diagnosis caused some worry in 85/110 patients (77 %, item 1, Fig. 1a). During follow-up 20/110 (18 %) thought about the lesion often (item 2). However, after the follow-up programme only 3/110 (3 %) patients reported significant impacts on their everyday life (item 4), and 15/110 (14 %) thought about the lesion sometimes, while 3/110 (3 %) thought about it often, or all the time (item 5).

#### Evaluation of the programme

Only 2/109 (2 %) reported that the termination of follow-up had made them feel more worried (item 3, Fig. 1b). In total, 11/110 (10 %) reported that their HRQL had been negatively impacted during the follow-up programme (item 6); however, only 4/110 (4 %) reported that they had experienced the programme as negative (item 7). The majority of patients were satisfied with the information received, but 21/109 (19 %) felt that the information about the adrenal lesion had been insufficient or very insufficient (item 8).

#### Assessment of information

The patients’ assessment of the information given (item 8) correlated with the reaction at termination of the programme (item 3: *r*_s_ = 0.33, *p* = 0.001), impact on everyday life (item 4: *r*_s_ = 0.22, *p* = 0.02), preoccupation during last month (item 5: *r*_s_ = 0.29, *p* = 0.002), and overall assessment of the programme afterwards (item 7: *r*_s_ = 0.30, *p* = 0.002). No differences in the patients’ assessments were seen between the participating clinical units.

### Responses to the hospital anxiety and depression scale and SF-36

In total, 12 respondents (11 %) scored as possible cases and 19 (18 %) as probable cases according to HADS-A. Sixteen (15 %) scored as possible cases and 16 (15 %) as probable cases according to HADS-D. There was considerable overlap in cases of anxiety and depression with 21 respondents scoring as possible or probable cases of both anxiety and depression.

**Fig. 2:**
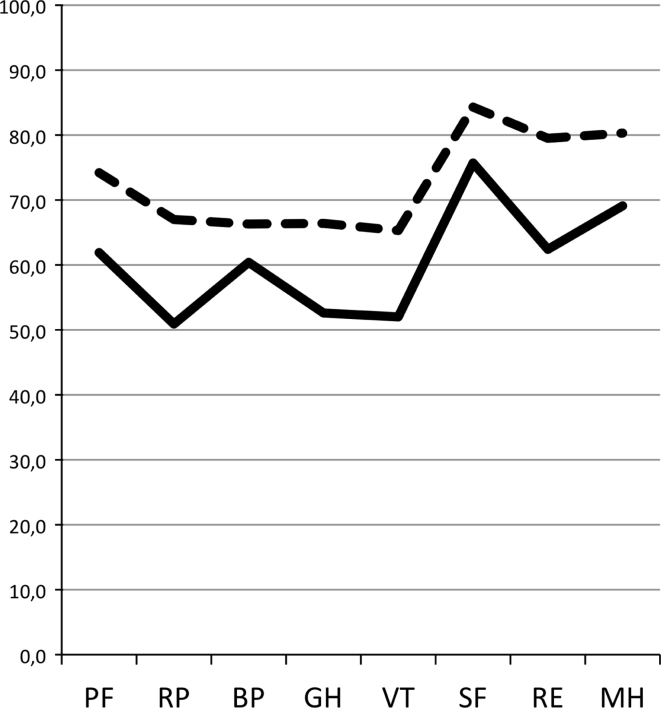
The short form-36—results for respondents versus age and gender-matched norms legend: means for individual domains for the respondents (*solid line*) and an age and sex-matched reference sample drawn from the Swedish norm database (*hatched line*). Differences were statistically significant in all domains (*p* = 0.00004–0.009), except BP (*p* = 0.085). *PF* physical functioning, *RP* role physical, *BP* bodily pain, *GH* general health, *VT* vitality, *SF* social functioning, *RE* role emotional, *MH* mental health

Respondents scored significantly lower than norms on all SF-36 domains, except bodily pain (Fig. 2). This finding was exclusively accounted for by respondents with one, or several co-morbidities (previous or concurrent extra-adrenal malignancy, cardiovascular disease or diabetes).

### Relationships between the AIIQ, HADS and SF-36

The preoccupation factor correlated with the HADS-A domain (*r*_s_ = −0.47, *p* < 0.001), and the SF-36 domains mental health (MH, *r*_s_ = 0.73, *p* < 0.001) and vitality (VT, *r*_s_ = −0.53, *p* < 0.001). Significant differences were seen between probable cases of anxiety and non-cases (HADS-A >10 vs. <8) regarding AIIQ items addressing preoccupation (items 1, 2, 4 and 5, *p* = 0.002–0.009), with probable cases scoring 0.61–0.85 units lower than non-cases. They also reported a greater impact on HRQL during follow-up (item 6, *p* = 0.006). However, no differences were seen regarding worry associated with the termination of the programme (item 3). Significant differences were also seen between probable cases of depression and non-cases (HADS-D >10 vs. <8) regarding impact on everyday life (item 4, *p* = 0.004), thoughts about the AI during the last month (item 5, *p* = 0.001), and general assessment of the programme after termination (item 7, *p* = 0.02), with probable cases of depression scoring 0.55–0.86 units lower than non-cases.

### Relationships between patient and tumour characteristics and health-related quality of life

Patient age was negatively correlated with the SF-36 domain physical functioning (PF, *r*_s_ = −0.34, *p* < 0.0003). Co-morbidity index also correlated with several domains of SF-36. Weaker, statistically not significant, associations were seen between co-morbidity and HADS-A (*r*_s_ = 0.18, *p* = 0.06), and HADS-D (*r*_s_ = 0.18, *p* = 0.06). No correlations were seen between HRQL and sex, tumour size, location (uni- or bilateral), UFC or 1 mg-DST cortisol levels, with the exception of UFC, where higher UFC levels correlated with better scoring on the SF-36 domain social functioning (SF, *r*_s_ = 0.21, *p* = 0.04) (Table 3). No significant differences were seen between HADS non-cases, possible and probable cases with regard to UFC or 1 mg-DST cortisol levels.

**Table 3:** Correlation analysis between patient and tumour characteristics and HRQL

	Sex	Age	Comorbidity index	Uni/bilateral	Size	UFC	1 mg-DST
AIIQ							
Preoccupation	0.083 (0.39)	0.036 (0.71)	−0.104 (0.28)	0.044 (0.65)	−0.037 (0.70)	−0.071 (0.49)	0.16 (0.15)
Evaluation	−0.002 (0.98)	0.049 (0.62)	0.013 (0.89)	−0.018 (0.85)	−0.081 (0.40)	−0.015 (0.88)	−0.12 (0.26)
HADS							
Anxiety	−0.066 (0.49)	−0.30 (0.76)	0.181 (0.062)	0.098 (0.31)	0.035 (0.72)	−0.13 (0.22)	−0.12 (0.29)
Depression	0.005 (0.96)	0.042 (0.67)	0.178 (0.065)	0.16 (0.099)	−0.082 (0.40)	−0.12 (0.24)	0.032 (0.79)
Short form-36							
Physical functioning	0.147 (0.13)	−0.335 (0.0003)*	−0.245 (0.010)*	−0.073 (0.45)	−0.075 (0.44)	−0.031 (0.76)	−0.035 (0.75)
Role physical	0.049 (0.612)	−0.166 (0.086)	−0.275 (0.004)*	−0.011 (0.91)	−0.078 (0.42)	0.069 (0.50)	0.044 (0.70)
Bodily pain	0.15 (0.12)	−0.064 (0.51)	−0.219 (0.023)*	−0.16 (0.11)	−0.103 (0.28)	0.005 (0.96)	0.026 (0.82)
General health	0.095 (0.32)	−0.056 (0.56)	0.323 (0.001)*	−0.12 (0.23)	−0.059 (0.54)	0.073 (0.47)	−0.095 (0.39)
Vitality	0.061 (0.53)	−0.036) (0.71)	−0.105 (0.28)	−0.182 (0.058)	−0.027 (0.78)	0.037 (0.72)	0.051 (0.65)
Social functioning	0.035 (0.72)	−0.060 (0.53)	−0.108 (0.27)	−0.11 (0.24)	−0.009 (0.93)	0.213 (0.035)*	−0.082 (0.46)
Role emotional	0.15 (0.12)	−0.134 (0.16)	−0.216 (0.025)*	−0.007 (0.95)	0.021 (0.83)	0.15 (0.15)	−0.060 (0.59)
Mental health	0.098 (0.31)	−0.022 (0.82)	−0.092 (0.34)	−0.166 (0.086)	0.044 (0.65)	0.13 (0.21)	0.003 (0.98)

Data presented as Spearman’s rho (*p* value), * *p* < 0.05

## Discussion

This study of patient-reported outcomes after completion of a 2-year follow-up programme for an incidentally discovered adrenal lesion without proven abnormal hormone production or malignancy suggests that the AI follow-up programme was well tolerated. Only 4 % experienced the follow-up programme as negative, and only 2 % reported increased worry after completing the programme. However, nearly 30 % were identified as having possible or probable anxiety or depression, and these patients also had a more negative experience of the programme.

We identified patients from a follow-up study in western Sweden [6, 7], in which all patients with AI detected at all radiology departments in our region (population 1.7 million) during an 18-month period were enrolled. The dropout rate during follow-up was low [6], and the present cohort may be regarded as representative for unselected patients diagnosed with AI in our region. The present study is, to our knowledge, the first that has attempted to directly assess patient reported outcomes of a follow-up programme in a large prospective, population-based series of conservatively managed AI patients. Owecki et al. [20] found increased anxiety levels and mild depression in a study of 26 polish patients with AI [20]. Brunaud et al. [21] measured the impact on HRQL of living with an AI using proxy surgeons’ ratings [21]. Kastelan et al. [22] recently reported on the HRQL of AI patients seen at a referral centre compared to an age and sex-matched control group.

In our study, 29 % of the respondents were identified as possible or probable cases of anxiety (HADS-A ≥8), and 30 % had possible or probable depression (HADS-D ≥8). Corresponding figures have been reported for patients with chronic diseases, such as coronary heart disease (HADS-A: 30–38 %, HADS-D: 15–50 %) [23–25]; diabetes type 2 (HADS-A: 20 %) [26]; chronic obstructive pulmonary disease (HADS-A: 27 %, HADS-D: 14 %) [27]; Parkinson’s disease (HADS-A: 44 %, HADS-D: 30 %) [28]; and after curative treatment for head and neck cancer (HADS-A: 16–28 %, HADS-D: 9–17 %) [29, 30].

Patients with AI are diagnosed as a consequence of work-up for an unrelated problem, and our study population has a high prevalence of co-existing morbidity (Table 1). Using a crude co-morbidity index there was a clear correlation between co-morbidity and worse HRQL in all physical domains of SF-36 as well as role emotional. Respondents also scored lower on nearly all domains of SF-36, reflecting a generally worse HRQL, compared with age and sex-matched norms from the general population. This is in agreement with the findings of Kastelan et al. [22].

Patients with probable anxiety (HADS-A >10) reported a greater impact on their HRQL during follow-up, but were not significantly more worried about the termination of the programme than were non-cases. The potential effect of subclinical hormone overproduction, e.g. subclinical hypercortisolism, is an intriguing issue [19]. However, in the present study UFC and 1 mg-DST cortisol levels, adrenal lesion size or bilaterality did not correlate significantly with any of the domains of AIIQ, HADS or SF-36. The only exception was the correlation between UFC levels and the SF-36 domain Social Function, where higher UFC levels were significantly associated with better Social Function, most likely a spurious correlation. HADS-A and HADS-D had a stronger, although not statistically significant (*p* = 0.06), relationship with co-morbidity index. This suggests that comorbidity, rather than a direct effect of the AI or the follow-up programme as such, was the main explanation for the levels of anxiety and depression seen in this patient group. In fact, only four respondents (4 %) rated their experience of the programme as negative. Still, the patient-reported measurements of the impact over time of the AI are retrospective and may be prone to recall bias, and definite conclusions about cause and effect cannot be drawn. Prospective studies are needed to study possible associations of anxiety and depression with tolerance to follow-up algorithms for AI.

Personalised and adequate patient information in a follow-up setting after a pathological test result has been shown to reduce anxiety [31]. We saw that a negative assessment of the given information significantly correlated with greater preoccupation with the AI after the programme. This is in line with the findings of Bell et al. [32], in a study of psychological adjustment to cervical screening, that patients reporting a high degree of initial worry at the abnormal test result were more likely to perceive the information they were given as inadequate and showed more concern at the time of the interview. Recently, Van Esch et al. [33] reported that the personality trait neuroticism and symptoms of depression and anxiety *prior* to a diagnosis of breast cancer were the most important predictors of HRQL 1 and 2 years after surgery. In a general setting, the perception of risk and worry are only weakly correlated [34], and patient-associated factors such as educational level [35, 36], living in urban or rural areas [36], number of children [36], and individual coping strategies [37] all correlate with psychological impacts. Still, focused counselling resources to patients with a high degree of anxiety at detection of AI may prevent unnecessary adrenalectomies and decrease negative impacts of follow-up.

Our study has limitations. Assessments were made only after completion of the follow-up programme. Hence, answers are retrospective in nature and no definite inferences regarding causality may be made. Due to the lack of appropriate instruments the AIIQ was specifically developed for this study. Although the AIIQ showed good face validity and construct validity (correlation between the preoccupation factor and HADS-A), the instrument needs to be further evaluated. Unfortunately, the Swedish normative database [15] did not allow matching for co-morbidities in the SF-36 comparison. Sociodemographic information was not collected, as it was felt that it might have reduced the response rate, thus further analyses by potentially relevant variables were precluded.

In summary, almost all patients in this cohort with incidentally discovered adrenal lesions reported satisfaction with the follow-up programme and were relieved or unconcerned when follow-up was ended. Although most were worried when the AI was detected, only a few remained worried during follow-up, and for these tailored counselling is suggested as a means to decrease negative impacts of follow-up. Overall, a 2-year follow-up programme for incidentally discovered adrenal lesions was well tolerated.
